# Thermal camouflage based on the phase-changing material GST

**DOI:** 10.1038/s41377-018-0038-5

**Published:** 2018-06-27

**Authors:** Yurui Qu, Qiang Li, Lu Cai, Meiyan Pan, Pintu Ghosh, Kaikai Du, Min Qiu

**Affiliations:** State Key Laboratory of Modern Optical Instrumentation, College of Optical Science and Engineering, Zhejiang University, Hangzhou, 310027 China

## Abstract

Camouflage technology has attracted growing interest for many thermal applications. Previous experimental demonstrations of thermal camouflage technology have not adequately explored the ability to continuously camouflage objects either at varying background temperatures or for wide observation angles. In this study, a thermal camouflage device incorporating the phase-changing material Ge_2_Sb_2_Te_5_ (GST) is experimentally demonstrated. It has been shown that near-perfect thermal camouflage can be continuously achieved for background temperatures ranging from 30 °C to 50 °C by tuning the emissivity of the device, which is attained by controlling the GST phase change. The thermal camouflage is robust when the observation angle is changed from 0° to 60°. This demonstration paves the way toward dynamic thermal emission control both within the scientific field and for practical applications in thermal information.

## Thermal camouflage: hidden in hot or cold

Thermal camouflage surfaces developed by Chinese researchers can be tailored to hide objects in front of different backgrounds. Traditional thermal camouflage comprises low-emissivity cloaks that lower the apparent temperature of vehicles or people to match their surroundings; however, objects can only be well-hidden when the background is one particular temperature. Qiang Li and co-workers at Zhejiang University in Hangzhou deposited a germanium-antimony-tellurium alloy onto gold film, before thermally annealing their samples at 200 °C. By varying the annealing time, the researchers prepared samples that were completely amorphous (randomly structured), completely crystalline (ordered), or intermediate states between the two. They found that the more crystalline samples had higher apparent temperatures, meaning the surfaces could be tailored to work at a range of background temperature. As well as benefitting military, such technology could allow better heat management during space travel.

## Introduction

Camouflage technology, which has the ability to hide or disguise an object (a person, an animal, or a piece of equipment) in the background^1^, has attracted increasing interest for many thermal applications. In terms of the physical mechanisms behind it, camouflage technology can be divided into two categories: color camouflage and thermal camouflage. Color camouflage is achieved by tuning light reflection or transmission to match the object’s appearance to the background in the visible or near-infrared range^2–4^. Many natural creatures such as cephalopods or chameleons can rapidly change their skin color to camouflage in this way^5,6^. The function of thermal camouflage, however, is to match the radiation temperature of a given object *T*_r_ (defined as the temperature at which a blackbody has an integrated radiance *B*^bb^(*T*_r_) equal to that of the object at temperature *T*, *B*^bb^(*T*_r_) = *B*(*T*)) with that of the background by controlling thermal emission. Therefore, thermal camouflage can befool the thermal imagers, which distinguish the object from the background based on their radiation temperature difference. Traditional thermal camouflage uses a low-emissivity coating, which reduces the object’s radiation temperature, to make the object blend into the background^7^. However, the emissivity of a traditional camouflage coating is fixed, and the objects can only be camouflaged in a fixed background temperature. Once the background temperature changes, the object can be easily discovered by the thermal imagers because of the radiation temperature difference between the object and the background. In addition, the observation angle robustness of thermal camouflage is an important factor for practical applications, as the targets can be discovered from wide observation angles. Therefore, thermal camouflage technology that can be used at varying background temperatures and wide observation angles needs to be explored.

Thermal camouflage with the ability to tune the thermal emissivity of the object can be used at varying background temperatures. Most of the methods to engineer emissivity and thermal emission are static^8–24^ and contribute to a number of applications, including radiative cooling^25–28^, thermophotovoltaics^29–35^, and sensing^36,37^. Recent works have extended emissivity control to dynamically reconfigurable materials, including graphene^38^, quantum wells^39^, doped zinc oxide^40^, and thermochromic metamaterials^41^. However, these works have not investigated their potential uses for creating thermal camouflage. It has, however, been demonstrated that the thermal emission of a device consisting of vanadium dioxide (VO_2_) can be controlled dynamically and it can therefore be used for thermal camouflage^42,43^. Moreover, thermal camouflage for a specific background temperature distribution can be created using natural materials^44^ or metamaterials based on transformation thermotics^45^. However, the ability to continuously camouflage objects either at varying background temperatures or for wide observation angles has not yet been experimentally explored.

In this study, a thermal camouflage device consisting of a Ge_2_Sb_2_Te_5_ (GST) film on top of a metal film is experimentally demonstrated. The phase-changing material GST has been used in various switchable optoelectronic devices^46–62^. Here we report the first thermal camouflage device incorporating the phase-changing material GST, which can camouflage at varying background temperatures and for wide observation angles. Continuous thermal camouflage is achieved by controlling the GST phase transition, during which the imaginary part of the permittivity of the GST increases, which, in turn, increases the emissivity. This thermal camouflage device presents several advantages as follows: (1) large background camouflage temperature range: thermal camouflage is experimentally demonstrated when the background temperature changes from 30 °C to 50 °C. (2) Robust with regard to the observation angle: the thermal camouflage is robust over a large range of observation angles, from 0° to 60°. (3) Size flexibility: the emissivity of the surface can be spatially tuned with a patterned layer of GST. We have demonstrated that the spatial emissivities of patterned GST-Au samples exhibit a spatial resolution of 500 μm (checkerboard pattern) and 100 μm (ZJU pattern). (4) Easy fabrication: the fabrication involves only simple film deposition. This thermal camouflage technology, based on phase-changing material, can be applied to dynamically control the thermal emission in fundamental science and significantly benefit a number of applications, including thermal camouflage and heat management for outer space.

## Materials and methods

### Fabrication of GST-based devices

To fabricate GST-Au devices, a 100 nm-thick Au film and subsequently a 350 nm GST film are deposited on a silicon substrate by high vacuum magnetron sputtering under room temperature. Both the GST thickness and the metallic material have been optimized to achieve a large camouflage temperature range (see Fig. S7 and Fig. S8). The GST alloy is composed of germanium (Ge), antimony (Sb), and tellurium (Te) with a deposition ratio of 2:2:5. The Ge component is DC sputtered with a power of 32 W, whereas the other two components (Sb and Te) are radio frequency (RF) sputtered with powers of 40 W and 60 W, respectively. The starting pressure and working pressure are set to 5 × 10^−4^ Pa and 0.1 Pa separately, to maintain the deposition rate at 0.53 nm s^−1^.

For the spatially resolved GST-Au devices, a 100 nm-thick Au film is deposited first. A 1.5 μm-thick photoresist (AR-P 5350) is then spun onto the Au film and annealed for 5 min at 105 °C. The photoresist is exposed to define a checkerboard pattern and a ZJU pattern using a double-sided mask aligner system (MA6–BSA). The photoresist is then developed in 1:6 AR 300-26/ Deionized (DI) water followed by rinsing in DI water. After development, a 350 nm-thick GST film is then deposited onto the sample by magnetron sputtering. The spatially resolved GST-Au devices are created after lift-off by ultrasonic processing in acetone for 1 min.

### Optical measurements

The infrared images are recorded by an infrared camera (FLIR S65). The spectral range of the infrared camera is between 7.5 μm and 13 μm. The spatial resolution of the infrared camera is 500 μm. The spectral radiance is measured by a Fourier transform infrared spectrometer (FTIR) with a room-temperature doped triglycine sulfate detector.

### Numerical simulations

The finite-difference time-domain method (FDTD Solutions v8.13, Lumerical) is used to compute the optical responses of the MIM thermal emitter. The relative permittivity of gold is obtained from Palik’s handbook. The relative permittivities of GST (2.5 µm–15 µm) are obtained experimentally from the fabricated GST films (Fig. S1). According to Kirchhoff’s law of thermal radiation, the absorptivities at different incident angles are equal to the emissivities at the corresponding observation angles. The simulated emissivities can be replaced by the simulated absorptivities, and the observation angles can be replaced by the incident angles.

## Results and discussion

The radiation temperature (*T*_r_) of an object recorded in infrared images is not equal to its real temperature (*T*) unless the object is a blackbody. The radiation temperature is related to several factors, which are included in the following equation:

1 $$T_r 	= C \times P_{obj}(T) = C{\int}_{\hskip -5pt\lambda _1}^{\lambda _2} {I_{obj}(\lambda ,T)d\lambda } \\ 	= C{\int}_{\hskip -5pt\lambda 1}^{\lambda _2} {\varepsilon _{obj}(\lambda )} \times \frac{{2\pi hc^2}}{{\lambda ^5}}\frac{1}{{e^{hc/\lambda k_BT} - 1}}d\lambda$$

where *T*_r_is the radiation temperature detected by an infrared (IR) camera, *C* is the specific constant of the IR camera, *P*_obj_ is the emitted power of the object detected by the IR camera, *I*_obj_ is the spectral radiance of the object, *ε*_obj_ is the emissivity of the object, and [λ_1_, λ_2_] is the spectral range of the IR camera (FLIR S65: 7.5 μm–13 μm). The radiation temperature of the object is directly related to the emitted power of the object *P*_obj_, which is determined by the emissivity of the object, the real temperature of the object, and the spectral range of the IR camera. Most natural environments, such as bare soil (emissivity *ε* = 0.91), grass (*ε* = 0.95), and shrub (*ε* = 0.98), have emissivity close to a blackbody. Furthermore, the temperature of the environmental background keeps changing while the temperature of the object, such as a vehicle, remains fixed under most circumstances. This makes the direct camouflage of a fixed object in a varying background difficult. By covering the object with camouflage coating with tunable emissivity, the radiation temperature of the object can be controlled to match that of the background so that an infrared camera cannot distinguish the object from the background. In this study, thermal camouflage is explored in the situation in which the object temperature is fixed at 60 °C and the background temperature is changed from 30 °C to 50 °C, to mimic the situation of an object at varying background temperatures. Thermal camouflage is not investigated at background temperatures below 30 °C in this study, as this would necessitate an additional cooling system.

**Fig. 1:**
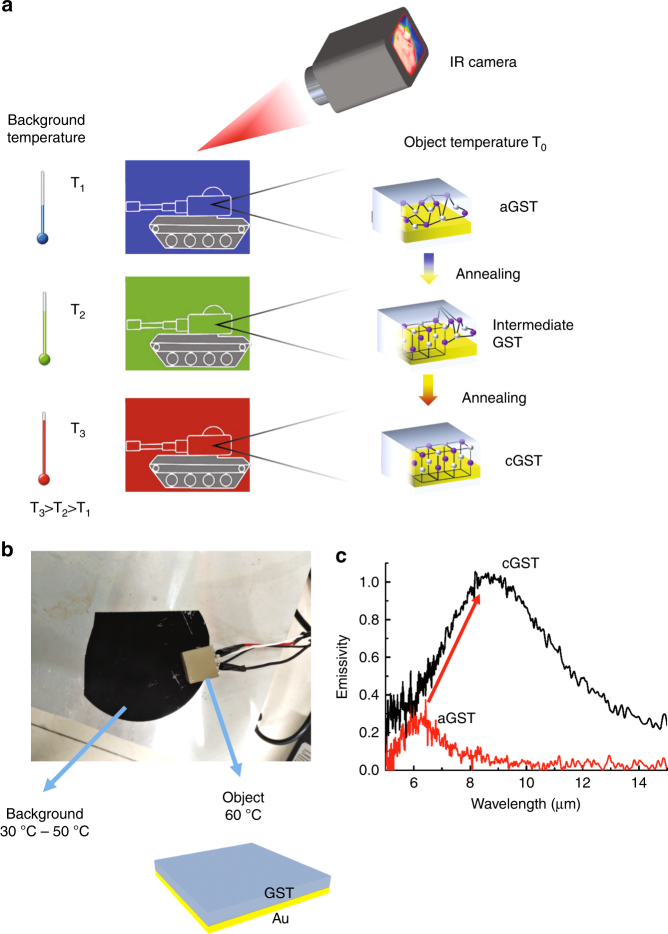
Concept of the thermal camouflage. **a** Illustration of the thermal camouflage with different background temperatures. The surface of the tank’s upper body is covered with a GST-Au double-layered film. Perfect camouflage can be achieved by changing the GST phase. **b** An image of the experimental setup. There are two heating stages in our setup. One is used to anneal the background (blackbody), with the temperature changing from 30 °C to 50 °C, and the other is used to anneal the object (emissivity-tunable sample) at a fixed temperature 60 °C. **c** The measured emissivities are collected at normal angle for aGST-Au and cGST-Au devices

The principle of thermal camouflage for different background temperatures based on the phase-changing material GST is illustrated in Fig. 1. The surface of an object (a tank here) is partly covered with a GST-Au double-layered film. When the background temperature increases from *T*_1_ to *T*_3_ (*T*_3_ > *T*_2_ > *T*_1_), the background color changes from blue (cold: a low radiation temperature) to red (hot: a high radiation temperature) in the thermal images. The surface of the tank’s upper body at a fixed temperature *T*_0_ (*T*_0_ > *T*_3_) can be made to blend into the background by controlling the thermal emission based on different GST phases. The as-deposited GST is in the amorphous phase (termed aGST) with a disordered atomic distribution. Intermediate GST (termed iGST) and crystalline GST (termed cGST) with a well-organized atomic distribution are obtained by annealing it at 200 °C on a hot plate for less than and greater than 60 s, respectively (see Fig. 1a). The intermediate GST consists of different proportions of amorphous and crystalline GST molecules. Different GST phases can be obtained by controlling the annealing time. GST has long been used in non-volatile memory, such as rewritable optical discs (in DVD and Blu-ray formats). It has been previously experimentally demonstrated that a GST film remains stable in a certain phase for years at room temperature^53,63^.

The relative permittivities of aGST and cGST are different due to their different atomic distributions. The imaginary part of the relative permittivity of aGST is negligible, whereas the imaginary part of the relative permittivity of cGST increases with the wavelength (Fig. S1). It can be concluded that aGST is transparent, whereas cGST is highly absorptive in the mid-infrared wavelength range. The GST film in the intermediate phases consists of different proportions of amorphous and crystalline molecules, and the effective permittivity of such a GST film can be estimated by the Lorentz–Lorenz relation^57^. For the aGST-Au device, there is no resistive loss in the aGST layer, and only resistive loss in the Au layer contributes to the thermal emission. For the cGST-Au device, resistive loss in both the cGST layer and the Au layer contributes to the thermal emission. This is why the aGST-Au device has a lower emissivity compared to the cGST-Au device (see Fig. 1c). The emissivities of aGST-Au and cGST-Au devices are measured using a FTIR. Further, the iGST-Au device has an emissivity between that of the aGST-Au and cGST-Au devices since the iGST film consists of different proportions of aGST and cGST. The radiation temperature of the GST-Au device also increases when GST is changed from aGST to cGST due to the increased emissivity.

The evolution of the X-ray diffraction (XRD) patterns of GST films with different annealing time (annealing temperature is fixed at 200 °C) is shown in Fig. S6. The as-deposited GST thin film has no diffraction peaks, indicating that it is in an amorphous state. Two peaks ((200) and (220)), which are the two most intense characteristic XRD peaks of the face-centered cubic (FCC) phase, appear when the annealing time is increased. It is observed that the diffraction intensity of these peaks ((200) and (220)) increases with annealing time, which indicates that crystallinity increases gradually with annealing time. The measured XRD results for different GST states are also in accordance with other reports^64,65^.

The experimental setup is shown in Fig. 1b. The blackbody (mimicking the background) temperature is changed from 30 °C to 50 °C, and the GST-Au device (mimicking the object) temperature is fixed at 60 °C. In this experiment, we use black soot as the background material. Black soot is generally regarded as a good reference to approximate a blackbody owing to its high wavelength-independent emissivity (approximately 0.97). Here, the black soot reference is produced by firing a rectangular stainless steel slice with a candle for 60 s. The object is annealed at 200 °C for different times to control the crystallization fraction of GST. The infrared images of the object and the background are recorded by the infrared camera simultaneously to evaluate the thermal camouflage ability of the device. As mentioned above, the emissivities of many natural environments are close to that of a blackbody and a blackbody is thus chosen as the background. The camouflage device is tested in the blackbody background to check the performance of the camouflage device in a natural environment-like situation. The samples used for camouflage are kept at a temperature higher than the background in this study, as a blackbody-like background is used. If a background with a low emissivity (such as gravel) is used, the sample temperature can be kept lower than that of the background.

**Fig. 2:**
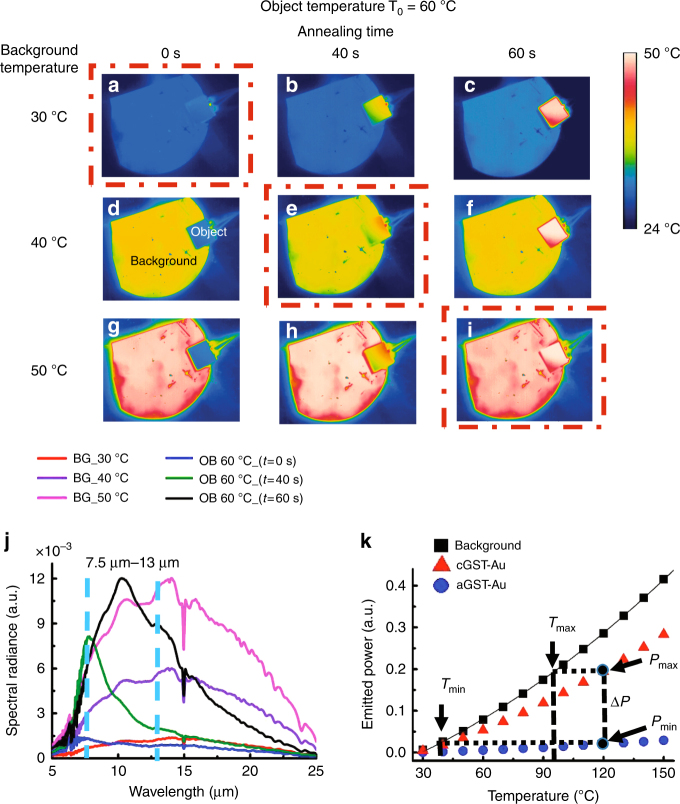
Infrared images and radiance spectra of the object and the background. **a**–**i** Infrared images are recorded by the infrared camera at different background temperatures (30 °C, 40 °C, and 50 °C) and a fixed object temperature (60 °C) after different annealing time. The object is annealed at 200 °C for 0 s, 40 s, and 60 s in order to obtain different GST crystallization fractions. **a**, **e**, and **i** are perfect thermal camouflage cases. **j** Thermal spectral radiance from the background at different heating temperatures and for different object annealing time. **k** Plot of the emitted power of the background, aGST-Au device, and cGST-Au device corresponding to their different temperatures. The emitted power tuning range Δ*P* (*P*_max_ − *P*_min_) is calculated from the difference between the emitted power of the cGST-Au device and the aGST-Au device

Infrared images are recorded to show the ability to achieve thermal camouflage at different background temperatures. When the background temperature is 30 °C, the radiation temperatures of both the object and the background are approximately 30 °C, and the object can hardly be distinguished from the background in the infrared image (Fig. 2a). To mimic different natural environments, the radiation temperature of the background is manipulated by increasing the real temperature from 30 °C to 50 °C with a step of 10 °C. The cold object can thereby be easily distinguished from the hot background since the radiation temperature of the object still remains at approximately 30 °C (Fig. 2d, g). The emissivity of the object is enhanced as the annealing time of the object is increased (Fig. S3). Therefore, the radiation temperature of the object increases and thermal camouflage is achieved. The radiation temperature of the object can be matched with the background temperature of 40 °C when the object is annealed at 200 °C for 40 s (Fig. 2e). The radiation temperature of the object can be further increased by annealing the object at 200 °C for 60 s and thermal camouflage can be achieved for the background temperature of 50 °C (Fig. 2i). As a result, thermal camouflage is achieved when the background temperature is changed from 30 °C to 50 °C.

The measured spectral radiance at different temperatures of the background (termed BG) and different annealing time of the object (termed OB) is shown in Fig. 2j. The radiance of the background is enhanced when the background temperature is increased from 30 °C to 50 °C. The radiance of the object at a fixed temperature of 60 °C also increases when the annealing time is increased. The emitted power of the background and the object is obtained by integrating the spectral radiance over the infrared camera’s spectral range from 7.5 μm to 13 μm. The emitted power of the background increases with background temperature (Fig. S4). The emitted power of the object can be tuned by manipulating the annealing time, and it can thereby be matched with that of the background at different temperatures. Therefore, the radiation temperatures of the object and the background can be close enough to fool the infrared imagers.

The emitted powers of the background, aGST-Au device, and cGST-Au device are shown in Fig. 2k. The emitted power is obtained by integrating the experimental spectral radiance over the spectral range from 7.5 μm to 13 μm. The emitted power tuning range Δ*P* (*P*_max_ − *P*_min_) is the difference between the emitted power of the cGST-Au and aGST-Au devices. If the emitted power of the background is in the range from *P*_min_ to *P*_max_, then thermal camouflage can be achieved by controlling the GST phase to match the emitted power of the GST-Au device with that of the background. The temperature of the background at which its emitted power is the same as *P*_max_ is *T*_max_. Similarly, the temperature of the background *T*_min_ corresponds to *P*_min_. The background camouflage temperature range Δ*T* is the difference between *T*_min_ and *T*_max_. The object can be camouflaged for a larger background temperature range by simply increasing the object temperature, as shown in Fig. S5. For example, when the object temperature is 30 °C, the background camouflage temperature range in which perfect camouflage can be achieved is from 28 °C to 30 °C. The background camouflage temperature range is from 41 °C to 119 °C when the object temperature is increased to 150 °C. This helps to camouflage the object in extreme environmental backgrounds with a large temperature range.

**Fig. 3:**
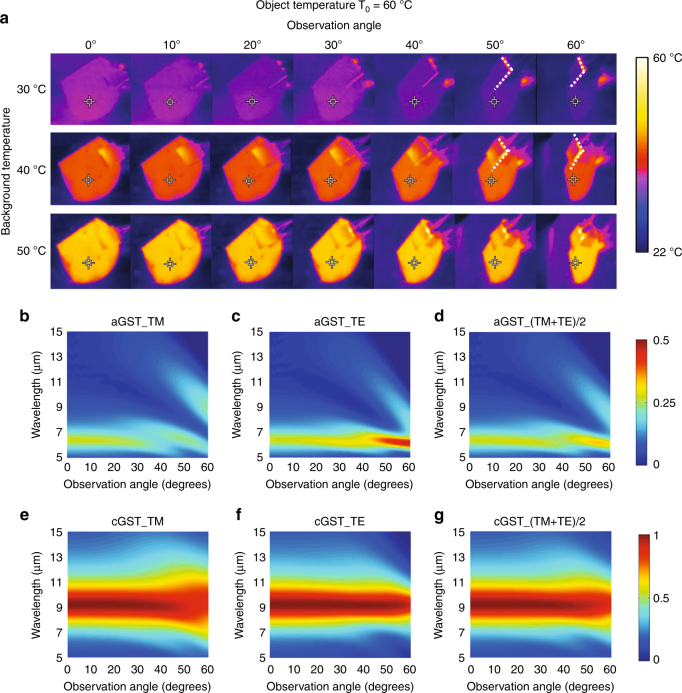
The robustness to the observation angle of the thermal camouflage. **a** The infrared images are recorded by the infrared camera at different observation angles ranging from 0° to 60°. The objects are aGST-Au, iGST-Au, and cGST-Au for the first, second, and third rows, respectively. The boundaries between the sample and the heater are marked by the white dotted lines in the images, which correspond to observation angles of 50° and 60°, and background temperatures of 30 °C and 40 °C. **b**–**g** Calculated observation angle dependence of the emissivities of aGST-Au and cGST-Au devices for TM, TE, and average of TM and TE polarizations

The robustness of thermal camouflage to the observation angle over a large range from 0° to 60° is presented in Fig. 3a. The first column of infrared images at the observation angle of 0° represents the perfect thermal camouflage cases shown in Fig. 2a, e, and i. When the observation angle is increased from 0° to 60°, the measured radiation temperature of the object remains almost the same as that of the background, indicating that the thermal camouflage device works well even at a large observation angles. The sample is actually annealed by a heater placed right beneath it. For small observation angles ( < 30°), the heater is hidden by the sample, as the view is from the top to the bottom (similar to a top view). For large observation angles ( > 30°), the heat below can be seen (similar to a side view). The heater has a lower emissivity than the sample and blackbody because of its metal surface. This is the reason why the objects look different at angles > 30° and angles < 30°. This thermal camouflage device has practical applications. Targets can be discovered from a wide range of observation angles, and therefore, the robustness of thermal camouflage to the observation angle is of paramount importance for the targets’ survivability. The thermal images of the device do not look very uniform, especially for the intermediate GST. This non-uniformity in the thermal images results from the gradual long-range variations in the film thickness and roughness, and from a slight inhomogeneity in the temperature distribution.

The emissivities of GST-Au devices at different observation angles are calculated to validate the robustness of the thermal camouflage over different observation angles. The infrared camera receives the thermal emission of both Transverse Electric (TE) and Transverse Magnetic (TM) polarizations. Therefore, we calculate the average emissivities of TM and TE polarizations for aGST-Au and cGST-Au devices, which are shown in Fig. 3d, g, respectively. These figures indicate that the emissivities remain independent of the observation angle (0° to 60°). This result confirms what we obtained from the infrared images. In Fig. 3a, the object in the first and third rows is in the aGST and cGST phases, respectively, and the object in the second row is in the iGST phase. For both aGST-Au and cGST-Au devices, the emissivities are insensitive to the observation angles for the TM, TE, and average of TM and TE polarizations (Fig. 3b-g). It can be concluded that the emissivities of the iGST-Au device are also insensitive to the observation angle because the iGST film can be regarded as a combination of aGST and cGST molecules. The experimental emissivities measured by FTIR also turn out to be independent of the observation angle, which is in agreement with the calculated emissivities (shown in Fig. S2).

**Fig. 4:**
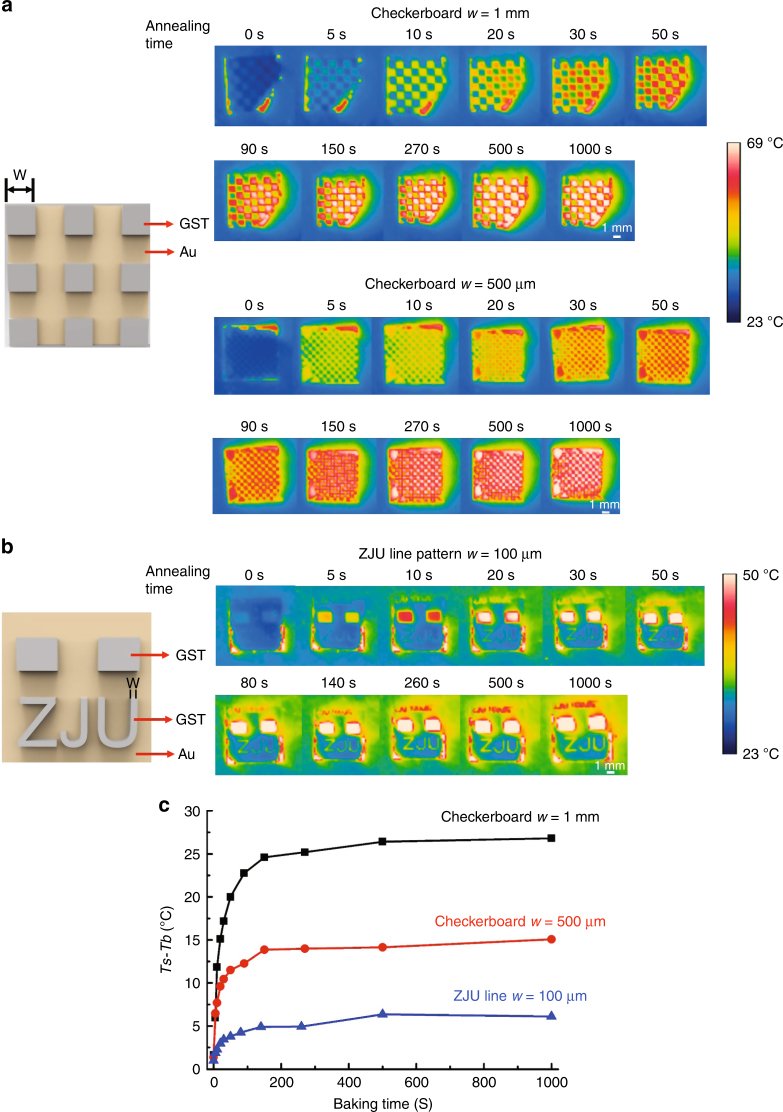
Spatial emissivities of patterned GST-Au devices. **a** Infrared images of the checkerboard pattern of GST-based devices with square size *w* of 1 mm and 500 μm. Infrared images are recorded using the infrared camera with a spatial resolution of 500 μm. **b** Infrared images of the ZJU pattern with line width *w* of 100 μm. The scale bar for (**a**) and (**b**) is 1 mm. **c** The difference of the radiation temperature (*Ts* − *Tb*) between the GST pattern and the background (Au film) in the checkerboard and ZJU patterns. Insets: schematic of the checkerboard pattern and ZJU pattern

To explore the camouflage limit for microscopic objects, the spatial emissivities of patterned GST-Au devices with different GST phases are studied further. The spatial shaping of the GST-Au devices’ emissivities can encode spatial information in infrared images by patterning the top layer GST. Spatially resolved infrared images of the checkerboard pattern and ZJU (abbreviated form of Zhejiang University) pattern are shown in Fig. 4a, b, respectively. For checkerboard patterns (*w* = 1 mm and *w* = 500 µm), the smallest squares cannot be clearly identified from the background (metal film) before annealing. This is because both the GST and the metallic background have a low radiation temperature. When the annealing time is increased gradually, the GST squares (red color) become prominent and can be distinguished from the metallic background (blue color) as the radiation temperature of the GST squares increases. When the square pixel size *w* is reduced to 500 µm, which is close to our infrared camera’s spatial resolution, the contrast of the adjacent areas decreases, but the squares can still be identified from the background. For the ZJU pattern, it can be identified even though the line width of the ZJU pattern is 100 μm. For the checkerboard pattern, the spatial camouflage resolution is limited by the resolution of the infrared camera. However, this limit is broken in case of the ZJU pattern, as it has much less disturbance from adjacent GST structures. Therefore, it can be concluded that the spatial camouflage resolution is limited by the surrounding structures and the infrared camera resolution. The two blocks in Fig. 4b are GST with the same thickness as the ZJU pattern. These two blocks are designed as markers for locating the ZJU pattern and for estimating the crystallization fraction of GST in the ZJU pattern by measuring their emission spectrum. The emission spectrum of the ZJU pattern cannot be directly measured, because the linewidth is too small.

The radiation temperature differences (*Ts* − *Tb*) between the GST film and the metallic background for different annealing time are shown in Fig. 4c. *Ts* and *Tb* are calculated by averaging the radiation temperature of ten arbitrarily selected points from GST and the metal background, respectively. The radiation temperature difference in the checkerboard pattern (*w* = 1 mm) increases from near 1 °C to 27 °C when the annealing time is increased from 0 s to 1000 s. For the square pixel size 500 µm, the radiation temperature difference can only increase up to 15 °C from 1 °C when the annealing time is increased from 0 s to 1000 s. The lower value of the radiation temperature difference for this checkerboard (*w* = 500 μm) can be attributed to the fact that the thermal camera spatial resolution is close to the size of the smallest GST squares. This leads to the lower average radiation temperature of the GST squares, whereas the average radiation temperature for the metal background is increased. The radiation temperature difference for the ZJU pattern increases up to approximately 6 °C from less than 1 °C when the annealing time of GST is increased from 0 s to 1000 s. The radiation temperature difference in the ZJU pattern is far less than that in the checkerboard pattern, because the ZJU line width (100 μm) is much less than the infrared camera’s spatial resolution (500 μm), and thermal emission from the ZJU line is averaged along with the adjacent low-emission metal background. This patterned GST on metal film can also be used to carry encoded spatial information, which can later be decoded using a thermal camera.

## Conclusions

In conclusion, a thermal camouflage device was investigated experimentally at varying background temperatures. It has been demonstrated that perfect thermal camouflage can be achieved when the background temperature changes continuously from 30 °C to 50 °C (the temperature of the object is fixed at 60 °C). The thermal camouflage is robust over a large range of observation angles, from 0° to 60°. Furthermore, it has been experimentally demonstrated that GST-Au devices with a top layer of patterned GST can encode spatial information, which can later be decoded using a thermal camera. This device also has the potential to work in a background changing from a high temperature to a low temperature by controlling the phase transition of GST from crystalline to amorphous (reamorphization). This reamorphization can be achieved by laser pulses (fs or ns)^46,47^ or electrical pulses^49,66^. This GST-based device manifests the ability to control thermal emissions dynamically and can significantly benefit new thermal camouflage technology.

## Electronic supplementary material

Supporting information

## References

[1] Han TC, Bai X, Thong JTL, Li BW, Qiu CW (2014). Full control and manipulation of heat signatures: cloaking, camouflage and thermal metamaterials. Adv. Mater..

[2] Phan L (2013). Reconfigurable infrared camouflage coatings from a cephalopod protein. Adv. Mater..

[3] Yu CJ (2014). Adaptive optoelectronic camouflage systems with designs inspired by cephalopod skins. Proc. Natl Acad. Sci. USA.

[4] Wang GP, Chen XC, Liu S, Wong C, Chu S (2016). Mechanical chameleon through dynamic real-time plasmonic tuning. ACS Nano.

[5] Mäthger LM, Denton EJ, Marshall NJ, Hanlon RT (2009). Mechanisms and behavioural functions of structural coloration in cephalopods. J. R. Soc. Interface.

[6] Teyssier J, Saenko SV, Marel D, Milinkovitch MC (2015). Photonic crystals cause active colour change in chameleons. Nat. Commun..

[7] Albertoni A. Long wave infrared metamaterials and nano-materials design, simulation, and laboratory test for target camouflage in the defence application. In *Proc. SPIE Electro-Optical and Infrared Systems: Technology and Applications VIII* Volume 8185, pp 818509 (SPIE, Prague, Czech Republic, 2011).

[8] Greffet JJ (2002). Coherent emission of light by thermal sources. Nature.

[9] Schuller JA, Taubner T, Brongersma ML (2009). Optical antenna thermal emitters. Nat. Photonics.

[10] Diem M, Koschny T, Soukoulis CM (2009). Wide-angle perfect absorber/thermal emitter in the terahertz regime. Phys. Rev. B.

[11] Mason JA, Smith S, Wasserman D (2011). Strong absorption and selective thermal emission from a midinfrared metamaterial. Appl. Phys. Lett..

[12] Liu XL (2011). Taming the blackbody with infrared metamaterials as selective thermal emitters. Phys. Rev. Lett..

[13] Makhsiyan M, Bouchon P, Jaeck J, Pelouard JL, Haïdar R (2015). Shaping the spatial and spectral emissivity at the diffraction limit. Appl. Phys. Lett..

[14] Roberts AS, Chirumamilla M, Thilsing-Hansen K, Pedersen K, Bozhevolnyi SI (2015). Near-infrared tailored thermal emission from wafer-scale continuous-film resonators. Opt. Express.

[15] Liu JJ (2015). Quasi-coherent thermal emitter based on refractory plasmonic materials. Opt. Mater. Express.

[16] Costantini D (2015). Plasmonic metasurface for directional and frequency-selective thermal emission. Phys. Rev. Appl..

[17] Park JH, Han SE, Nagpal P, Norris DJ (2016). Observation of thermal beaming from tungsten and molybdenum bull’s eyes. ACS Photonics.

[18] Liao CY (2016). Quasi-coherent thermal radiation with multiple resonant plasmonic cavities. Appl. Phys. Lett..

[19] Huang WL, Hsiao HH, Tang MR, Lee SC (2016). Triple-wavelength infrared plasmonic thermal emitter using hybrid dielectric materials in periodic arrangement. Appl. Phys. Lett..

[20] Ilic O (2016). Tailoring high-temperature radiation and the resurrection of the incandescent source. Nat. Nanotechnol..

[21] Liu BA, Gong W, Yu BW, Li FP, Shen S (2017). Perfect thermal emission by nanoscale transmission line resonators. Nano Lett..

[22] Yang ZY (2017). Narrowband wavelength selective thermal emitters by confined tamm plasmon polaritons. ACS Photonics.

[23] Zhou M (2015). Analog of superradiant emission in thermal emitters. Phys. Rev. B.

[24] Zhang X, Liu H, Zhang ZG, Wang Q, Zhu SN (2017). Controlling thermal emission of phonon by magnetic metasurfaces. Sci. Rep..

[25] Raman AP, Anoma MA, Zhu LX, Rephaeli E, Fan SH (2014). Passive radiative cooling below ambient air temperature under direct sunlight. Nature.

[26] Zhu LX, Raman AP, Fan SH (2015). Radiative cooling of solar absorbers using a visibly transparent photonic crystal thermal blackbody. Proc. Natl Acad. Sci. USA.

[27] Hsu PC (2016). Radiative human body cooling by nanoporous polyethylene textile. Science.

[28] Zhai Y (2017). Scalable-manufactured randomized glass-polymer hybrid metamaterial for daytime radiative cooling. Science.

[29] Nagpal P, Han SE, Stein A, Norris DJ (2008). Efficient low-temperature thermophotovoltaic emitters from metallic photonic crystals. Nano Lett..

[30] Chan WR (2013). Toward high-energy-density, high-efficiency, and moderate-temperature chip-scale thermophotovoltaics. Proc. Natl Acad. Sci. USA.

[31] Lenert A (2014). A nanophotonic solar thermophotovoltaic device. Nat. Nanotechnol..

[32] Rinnerbauer V (2014). Metallic photonic crystal absorber-emitter for efficient spectral control in high-temperature solar thermophotovoltaics. Adv. Energy Mater..

[33] Zhou J, Chen X, Guo LJ (2016). Efficient thermal-light interconversions based on optical topological transition in the metal-dielectric multilayered metamaterials. Adv. Mater..

[34] Dyachenko PN (2016). Controlling thermal emission with refractory epsilon-near-zero metamaterials via topological transitions. Nat. Commun..

[35] Asano T (2016). Near-infrared-to-visible highly selective thermal emitters based on an intrinsic semiconductor. Sci. Adv..

[36] Miyazaki HT (2014). Dual-band infrared metasurface thermal emitter for CO_2_ sensing. Appl. Phys. Lett..

[37] Lochbaum A (2017). On-chip narrowband thermal emitter for mid-IR optical gas sensing. ACS Photonics.

[38] Brar VW (2015). Electronic modulation of infrared radiation in graphene plasmonic resonators. Nat. Commun..

[39] Inoue T, De Zoysa M, Asano T, Noda S (2014). Realization of dynamic thermal emission control. Nat. Mater..

[40] Coppens ZJ, Valentine JG (2017). Spatial and temporal modulation of thermal emission. Adv. Mater..

[41] Liu XY, Padilla WJ (2016). Thermochromic infrared metamaterials. Adv. Mater..

[42] Kats MA (2013). Vanadium dioxide as a natural disordered metamaterial: perfect thermal emission and large broadband negative differential thermal emittance. Phys. Rev. X.

[43] Xiao L (2015). Fast adaptive thermal camouflage based on flexible VO_2_/graphene/CNT thin films. Nano Lett..

[44] Yang TZ (2015). Invisible sensors: simultaneous sensing and camouflaging in multiphysical fields. Adv. Mater..

[45] Li Y, Bai X, Yang TZ, Luo HL, Qiu CW (2018). Structured thermal surface for radiative camouflage. Nat. Commun..

[46] Gholipour B, Zhang JF, MacDonald KF, Hewak DW, Zheludev NI (2013). An all-optical, non-volatile, bidirectional, phase-change meta-switch. Adv. Mater..

[47] Michel AKU (2014). Reversible optical switching of infrared antenna resonances with ultrathin phase-change layers using femtosecond laser pulses. ACS Photonics.

[48] Rios C, Hosseini P, Wright CD, Bhaskaran H, Pernice WHP (2014). On-chip photonic memory elements employing phase-change materials. Adv. Mater..

[49] Hosseini P, Wright CD, Bhaskaran H (2014). An optoelectronic framework enabled by low-dimensional phase-change films. Nature.

[50] Schlich FF, Zalden P, Lindenberg AM, Spolenak R (2015). Color switching with enhanced optical contrast in ultrathin phase-change materials and semiconductors induced by femtosecond laser pulses. ACS Photonics.

[51] Tittl A (2015). A switchable mid-Infrared plasmonic perfect absorber with multispectral thermal imaging capability. Adv. Mater..

[52] Hira T (2015). All-optical switching of localized surface plasmon resonance in single gold nanosandwich using GeSbTe film as an active medium. Appl. Phys. Lett..

[53] Ríos C (2015). Integrated all-photonic non-volatile multi-level memory. Nat. Photonics.

[54] Wang Q (2015). Optically reconfigurable metasurfaces and photonic devices based on phase change materials. Nat. Photonics.

[55] Yoo S, Gwon T, Eom T, Kim S, Hwang CS (2016). Multicolor changeable optical coating by adopting multiple layers of ultrathin phase change material film. ACS Photonics.

[56] Rudé M (2016). Ultrafast and broadband tuning of resonant optical nanostructures using phase-change materials. Adv. Opt. Mater..

[57] Chu CH (2016). Active dielectric metasurface based on phase-change medium. Laser Photonics Rev..

[58] Li PN (2016). Reversible optical switching of highly confined phonon-polaritons with an ultrathin phase-change material. Nat. Mater..

[59] Du KK (2017). Control over emissivity of zero-static-power thermal emitters based on phase-changing material GST. Light Sci. Appl..

[60] Mkhitaryan VK (2017). Tunable complete optical absorption in multilayer structures including Ge_2_Sb_2_Te_5_ without lithographic patterns. Adv. Opt. Mater..

[61] Yin XH (2017). Beam switching and bifocal zoom lensing using active plasmonic metasurfaces. Light Sci. Appl..

[62] Cao T, Wei CW, Simpson RE, Zhang L, Cryan MJ (2014). Broadband polarization-independent perfect absorber using a phase-change metamaterial at visible frequencies. Sci. Rep..

[63] Lee SH, Jung Y, Agarwal R (2007). Highly scalable non-volatile and ultra-low-power phase-change nanowire memory. Nat. Nanotechnol..

[64] Yu X (2017). Improved multi-level data storage properties of germanium-antimony-tellurium films by nitrogen doping. Scr. Mater..

[65] Fan T (2017). The crystallization behavior of amorphous Ge_2_Sb_2_Te_5_ films induced by a multi-pulsed nanosecond laser. Semicond. Sci. Tech..

[66] Hu YF (2015). Ge_2_Sb_2_Te_5_/Sb superlattice-like thin film for high speed phase change memory application. Appl. Phys. Lett..

