# Supplementary material for: Thermal camouflage based on the phase-changing material GST
Source: Light Sci Appl. 2018 Jun 27;7:26. doi: 10.1038/s41377-018-0038-5 (PMC6107009; doi:10.1038/s41377-018-0038-5)
Supplement: Supplementary file 1 — Supporting information [file 41377_2018_38_MOESM1_ESM.docx]

Supplementary Information

**Thermal camouflage based on the phase-changing material GST**

Yurui Qu, Qiang Li*, Lu Cai, Meiyan Pan, Pintu Ghosh, Kaikai Du and Min Qiu

State Key Laboratory of Modern Optical Instrumentation, College of Optical Science and Engineering, Zhejiang University, 310027, Hangzhou, China

E-mail: [qiangli@zju.edu.cn](mailto:qiangli@zju.edu.cn)

**1. The relative permittivities of aGST and cGST.**


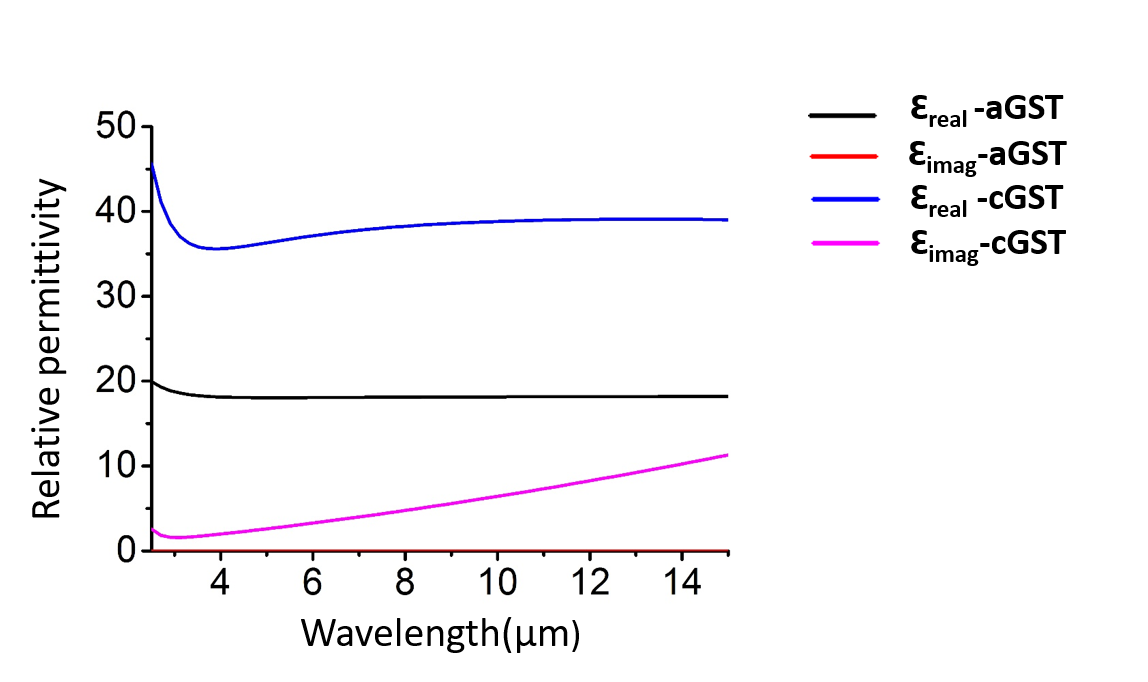


**Figure S1.** The relative permittivity plots for aGST and cGST.

**2. The measured and calculated emissivities of aGST-Au and cGST-Au devices at different observation angles.**


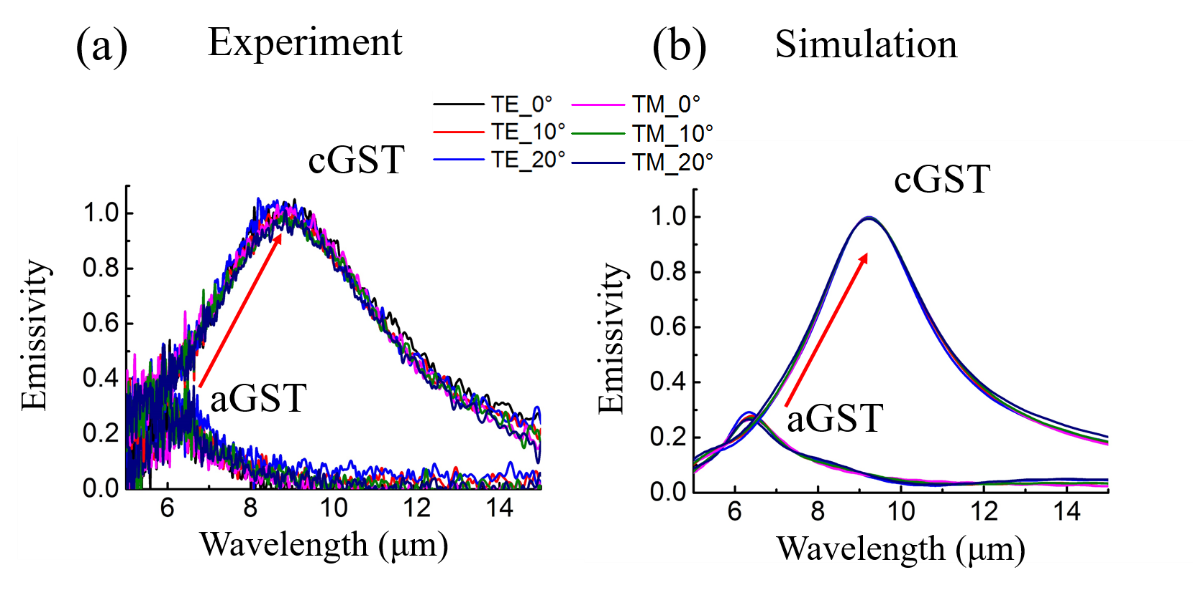


**Figure S2.** The measured **(a)** and calculated **(b)** emissivities of aGST-Au and cGST-Au devices at different observation angles for TE and TM polarization.

**3. The experimental emissivities of GST-Au devices for different annealing time.**


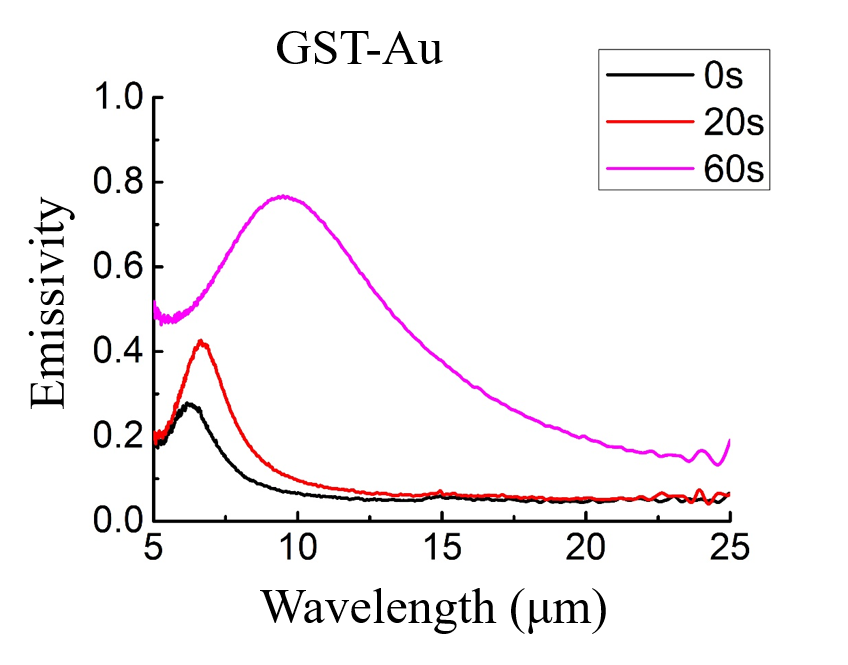


**Figure S3.** Experimental emissivities of GST-Au devices for different annealing time. The thickness of GST film for GST-Au devices is 350 nm.

**4. The emitted power of the object and background.**

**
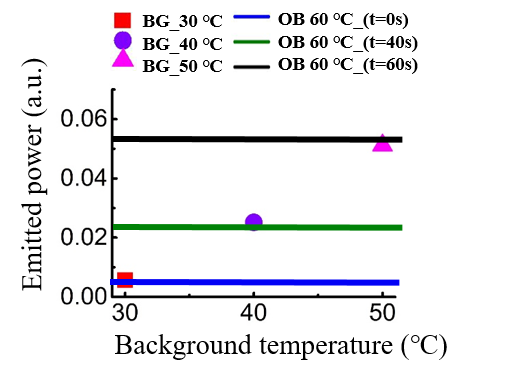
**

**Figure S4.** The emitted power of the object and background can be obtained from the integral of spectral radiance on the interval [7.5 μm, 13 μm]

**5. The background temperatures at which perfect camouflage can be achieved at different object temperatures.**

**
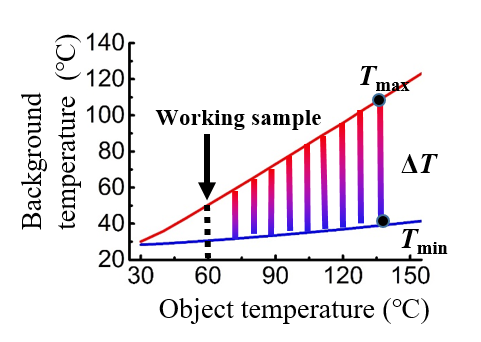
**

**Figure S5.** The background temperatures at which perfect camouflage can be achieved at different object temperatures. Red line represents cGST-Au device and blue line represents aGST-Au device. ΔT is the background camouflage temperature range where perfect camouflage can be achieved at different object temperatures.

**6. XRD patterns of GST films with different annealing time at the annealing temperature of 200 ℃.**


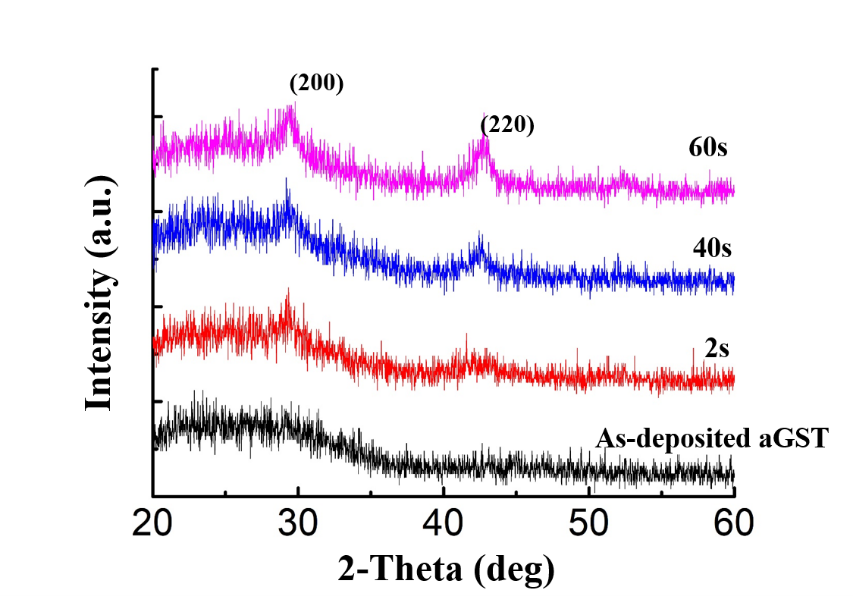


**Figure S6.** Evolution of XRD patterns of GST films with different annealing time. The annealing temperature is fixed at 200 °C.

**7. The emissivities for aGST-Au and cGST-Au devices with different GST thicknesses.**

With the increased GST thickness, the emission peak shifts to longer wavelength for both aGST and cGST sample (see Fig. S7). The spectral response range of the infrared camera is between 7.5 μm to 13 μm in this experiment. Our goal is to achieve high emission for cGST sample and low emission for aGST sample, so that a large camouflage temperature range can be realized. For 250 nm GST thickness, the emission peak of the cGST sample is below 7.5 μm. For both 350 nm and 450 nm GST thickness, the samples have almost the same high emission power in the 7.5 μm -13 μm range at cGST states. However, the aGST sample with 350 nm GST thickness shows lower emission power (red line). Therefore, the 350 nm thickness is chosen for GST layer in this paper.


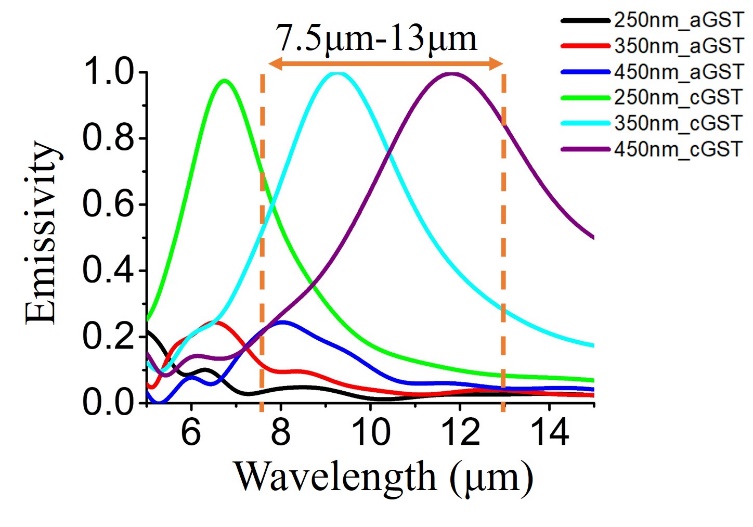


**Figure S7.** The simulated emissivities at normal angle for aGST-Au and cGST-Au devices with different GST thicknesses.

**8. The simulated emissivities for aGST-based and cGST-based devices with different materials of the bottom metal layer Au, Ag and Pt.**

Figure S8 provides the simulated emissivities for aGST-based and cGST-based devices with different materials of the bottom metal layer (Au, Ag and Pt). The aGST-Pt device shows higher emissivity than aGST-Au and aGST-Ag devices between 7.5 μm and 13 μm, which could reduce the camouflage temperature range. The GST-Au and GST-Ag devices show similar emissivities, but Ag is comparatively instable at high annealing temperature. Therefore, Au is chosen as the material of the bottom metal layer.

**
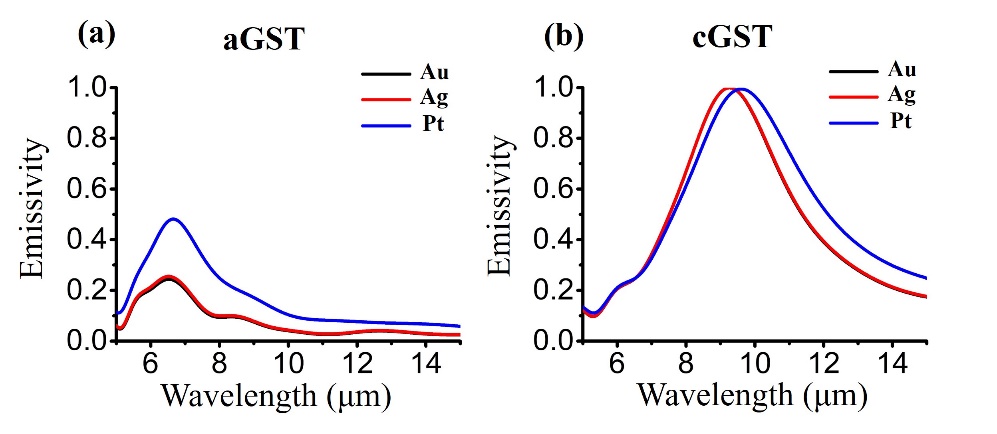
**

**Fig. S8** The simulated emissivities for (a) aGST-based and (b) cGST-based devices with different materials of the bottom metal layer Au, Ag and Pt. The emissivity curve of cGST-Au device (black line) totally coincides with that of cGST-Ag device (red line) in Fig. S8 (b).

**9. The amplitude of magnetic field distribution in the Au-GST–Au (MIM) structure and GST-Au double layer structure with a 350-nm-thick GST.**

The strong emission peak of the MIM structure results from the typical magnetic resonance (including the fundamental and high-order magnetic resonances). The magnetic field of the magnetic resonance is confined to the intermediate GST film right below the top Au nanodisks (see Fig. S9 (a)). The emission peak of the double-layer structure comes from destructive interference in the GST layer. The magnetic field increases as light propagates into the GST layers and is quite strong at the GST-Au interface (see Fig. S9 (b)).


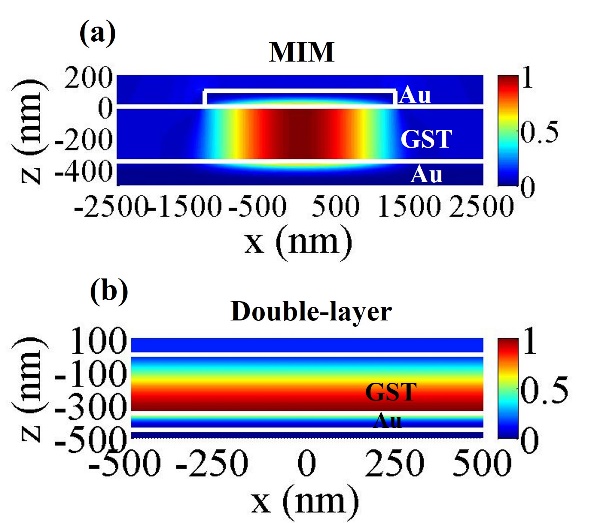


**Fig. S9** The amplitude of magnetic field distribution in (a) the Au-GST–Au MIM structure and (b) GST-Au double layer structure with a 350-nm-thick GST.

**10. The simulated emissivities at normal angle for MIM and double-layer devices.**

The MIM structure and double-layer structure show different emission performance, as shown in Fig. S10. The spectral range of the infrared camera used in this experiment is 7.5 μm-13 μm. For cGST-based device, the MIM and double layer structure have similar thermal emission power in camera’s spectral range. However, the MIM structure has higher emission power for aGST-based device than double layer structure. High emission peak comes from the magnetic resonance based on the MIM structure. Therefore, the double layer structure has larger camouflage temperature range than MIM structure. This is the main reason why we chose double-layer structure in this experiment.


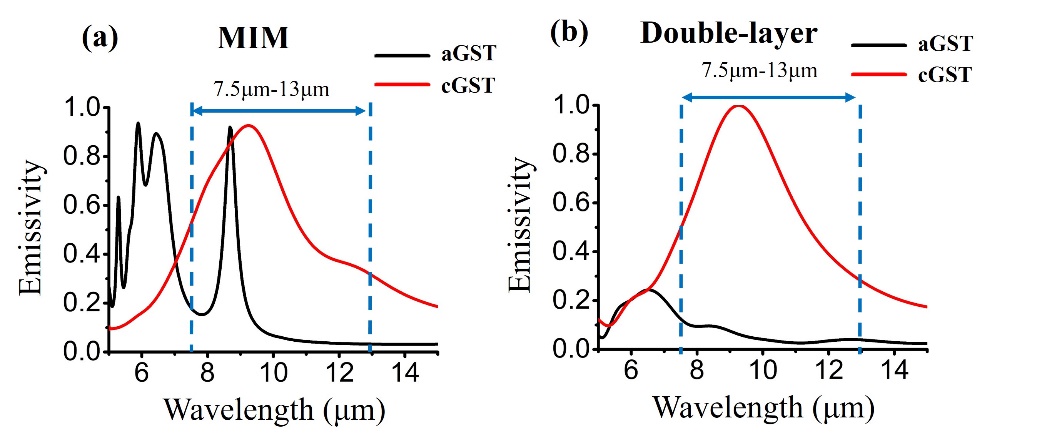


**Fig. S10** The simulated emissivities at normal angle for (a) MIM and (b) double-layer devices.
